# A Report of Two Incomplete Ovariotomies—One Fatal

**Published:** 1889-08

**Authors:** John J. Buchanan

**Affiliations:** Pittsburgh, Pa.


### A REPORT OF TWO INCOMPLETE OVARIOTOMIES—ONE FATAL.

BY JOHN J. BUCHANAN, M. D., PITTSBURGH, PA.

Read at Allegheny County Medical Society, May 21, 1889.

In two cases of ovarian tumor in which I have been called to operate, I have been unable to complete the operation on account of the extent, locality and tenacity of the adhesions. The first operation was performed on July 13th, 1885, and resulted in the death of the patient on the 32d day. The second was done five weeks ago, and the patient is now convalescent. These have been far the most instructive of the cases upon which I have operated, and a brief history of them may be of interest.

*Case 1.*—Patient aged 20 years, married one year, was of slight build and considerably emaciated. Two and a half months before operation the abdomen began to perceptibly enlarge. In one month the enlargement was so considerable and her distress so great that her attendant drew off a portion of the fluid by aspiration. Refilling rapidly occurred, and at the end of another month her condition urgently demanded operation. The tumor was immovable by external or combined examination and the uterus was firmly wedged in the pelvis. When the abdominal wall was incised, the peritoneum could not be identified, and the tumor was opened and its contents evacuated. The cyst was found to be universally adherent, and so tenacious were the adhesions that, in the effort to separate the cyst from the abdominal wall, the former was torn to shreds. The intestines were tightly attached above and the bladder below. In the effort to release the vesical adhesions a slight rent was made in the fundus of the bladder. This was sutured and thereafter gave no trouble. Enucleation was tried and failed. The hand in the cyst found it plastered over and tightly adherent to the pelvic walls and viscera.

After the most persistent endeavors to release the cyst it was reluctantly decided to stitch it to the abdominal wound and drain. Glass tubes were placed in the sac and in the peritoneal cavity. The peritoneal tube was allowed to remain seven days, and on its removal the opening filled up at once; the tube in the cyst remained eleven days, and was then replaced by one of soft rubber. During the first three days after the operation, the discharge from the tube in the cyst was odorless; on the fifth day a putrefactive odor was observed, due probably to decomposition of blood-clot in the cyst cavity. Despite frequent washings with carbolic solution this odor persisted till, on the 12th day, fæcal matter was found in the discharges from the cyst cavity. From this time forward her alvine discharges were almost entirely through the fistula. The cyst cavity did not collapse; exploration with the finger and by reflected light showed the cyst wall in a state of gray slough with granulations pushing through; the size of the cavity about six fluid ounces; the wall hard, rugous and insensible, except in the right iliac fossa, where the surface was soft, velvety and tender to the touch. She had a severe chill on the 17th day, and gradually declined in strength till the 32d day, when she died.

No *post mortem* examination was made.

*Case 2.*—Patient of Drs. Pershing and Rigg, of Wilkinsburg; aged 35 years, married; the mother of two children, the younger nine months old. At the time of her last confinement she was attended by Dr. Pershing, and nothing abnormal was developed. Two months later she discovered an immovable growth in her right ovarian region, about the size of a small egg. This increased but little during the next four months. A few days before operation it was seen for the first time, and was found to be about the size of an adult head. During the two weeks previous it had doubled in size. Her temperature was 103°; she was confined to bed, suffering considerably. Her attendants used the hypodermic needle for purposes of diagnosis and secured a yellow puriform liquid, which subsequently congealed. Microscopical examination showed it to be made up largely of fat globules with a few leucocytes. The cervix uteri was crowded forward in the pelvis and perfectly immovable. A diagnosis of adherent dermoid ovarian cyst was based on the rapid increase in size, the inflammatory symptoms and the nature of the fluid withdrawn. An immediate operation was decided on and performed with the assistance of Drs. Werder, Pershing and Rigg. Identification of tissues was very difficult. The great omentum was much hypertrophied and infiltrated and attached below to the fundus of the bladder; it was tightly adherent in front to the abdominal wall and behind to the cyst. The cyst was universally adherent; not one square inch of free surface could be found; the adhesions were of the firmest character. The great omentum was loosened from the cyst, secured with clamp forceps above and an elastic ligature below, and cut transversely between. A sound was now passed into the bladder and it was found that the elastic ligature embraced not only the great omentum, but also a portion of the fundus of the bladder. The ligature was at once loosened and the cut omental surfaces seared with the Paquelin cautery. The cyst was forcibly loosened over a considerable surface on either side and ruptured in the effort. The fluid evacuated was of the same nature as that withdrawn by the needle, and there was also secured a small bunch of long black hair, thus verifying the diagnosis. As soon as the rupture occurred the cyst was freely opened in front. There were two distinct cavities, the larger below. It was impossible to prevent the fluid from entering the peritoneal cavity. Five or six gallons of boiled water was used to wash out the peritoneal and cyst cavities. What was evidently a tooth could be felt in the bottom of the cyst wall. After every justifiable effort had apparently been made to overcome the intestinal adhesions, a plait was taken in the cyst wall on either side and the margins of the opening in the cyst stitched to the lower part of the abdominal incision, a glass drain being left in the peritoneal cavity and one in the cyst.

The peritoneal tube was removed at the end of twenty-four hours, and the tube in the sac was replaced by a rubber one on the third day. Suppuration occurred in and about the cyst, but the highest temperature during the first ten days was 100 2-5°. Some obstruction to the discharge of pus from the cavity between the bladder and the cyst occurred during the third week and caused an elevation of temperature. In other respects her recovery was uneventful. The sac collapsed and sloughed. It was washed out three times a day with boiled water, and occasionally with sublimate solution. During the first two weeks there was some discharge of oily matter with the pus, but none since. She is now up and about the house, and the discharge from the sinus is diminishing.

In connection with the history of these two cases, the question arises—could these cysts have been removed? Mr. Lawson Tait at the close of last year said :* "I am now in position to say that no cystic tumor of the abdomen exists which cannot be removed. . . . In my second series I have only to plead guilty to six uncompleted operations in 1,000, whereas, in my first series, the number was thirty, with the same mortality in each—50 per cent. Even in these six I know now, for *post mortem* examination revealed the fact, that I could have finished the operations in at least two, perhaps in three, if I had screwed my courage up to a still harder strain, and I blame myself accordingly."

Mr. Tait, in his work on "Diseases of the Ovaries,"† says: "Whenever, in an exploratory incision, the bladder is found pulled up and spread over the front of the tumor for a considerable distance, the proceeding may at once be brought to a conclusion, for it may be regarded as perfectly certain that the tumor cannot be removed."

Olshausen‡ says: "At the present time it may be claimed that adhesions never make the operation impossible. . . . But when the connection of the tumor with adjacent organs, especially the rectum, is unusually firm, and when the papillary formations of the tumor have grown into the wall of the organs, complete extirpation will be impossible. How rare this is in reality is shown by Schrœder's dictum that nearly every ovarian tumor may be extirpated. Tumors which he could not remove two

*General Summary of Conclusions from a Second Series of One Thousand Consecutive Cases of Abdominal Section.—*British Medical Journal, Nov.* 17, 1888.

†The Pathology and Treatment of Diseases of the Ovaries. New York: Wm. Wood & Co.

‡Disease of the Ovaries. By R. Olshausen. New York: Wm. Wood & Co.

years ago, he now extirpated completely in a second laparotomy."

Kaltenbach* says: "And despite all our progress in *technique*, cases remain in which the operation cannot be completed, because the connections of the tumor with surrounding parts are too firm and extensive, or are imperfectly defined. . . . In cystic tumors we may confine ourselves to partial extirpation, and stitch the pelvic segment of the tumor into the lower angle of the wound."

Fritsch† says: "Should the adhesions in the lesser pelvis be firm, it would be exceedingly dangerous to loosen them. . . . Shrœder obtained the best results by cutting off the upper part of the cyst and sewing the rest into the abdominal wound. The sac, thus separated from the peritoneum, rapidly diminished." My friend, Dr. Howard A. Kelly, of Philadelphia, who passed through the city this morning, tells me that he has never yet encountered an ovarian cyst which he could not remove.

On the other hand, Dr. Skene Keith, in his second series of fifty ovariotomies had two incomplete operations, with one death. Dr. John Homans, in 290 ovariotomies, stitched the cyst to the skin in eight cases; all recovered. Mr. H. C. Cameron, in twenty-eight cases, had two incomplete operations, with one death. Terrier, in twenty-fiive cases recently reported, had five incomplete operations, with two deaths. It is evident from these quotations and statements that incomplete operations occasionally happen in the practice of the best operators, but as their experience grows, such cases get less frequent, even to the point of disappearance; but when they do happen the mortality approximates 50 per cent.

My own impression is, that if a cyst, firmly and extensively adherent to intestines, bladder and pelvic floor, is encountered by an operator who has not a most extensive experience and extraordinary skill, and if he feels that persistence in attempts at removal will result in the rupture of intestines, bladder or iliac vessels, his patient is much safer with the incomplete operation.

---

*A Handbook of General and Operative Gynecology. By Dr. A. Hegar and Dr. R. Kaltenback, New York: Wm. Wood & Co.

†The Diseases of Women. By Heinrich Fritsch. New York: Wm. Wood & Co.

